# A Unique Human-Fox Burial from a Pre-Natufian Cemetery in the Levant (Jordan)

**DOI:** 10.1371/journal.pone.0015815

**Published:** 2011-01-26

**Authors:** Lisa A. Maher, Jay T. Stock, Sarah Finney, James J. N. Heywood, Preston T. Miracle, Edward B. Banning

**Affiliations:** 1 Leverhulme Centre for Human Evolutionary Studies, University of Cambridge, Cambridge, United Kingdom; 2 Department of Earth Sciences, University of Cambridge, Cambridge, United Kingdom; 3 Department of Paediatrics, University of Cambridge, Cambridge, United Kingdom; 4 Department of Archaeology, University of Cambridge, Cambridge, United Kingdom; 5 Department of Anthropology, University of Toronto, Toronto, Canada; University of Oxford, United Kingdom

## Abstract

New human burials from northern Jordan provide important insights into the appearance of cemeteries and the nature of human-animal relationships within mortuary contexts during the Epipalaeolithic period (c. 23,000–11,600 cal BP) in the Levant, reinforcing a socio-ideological relationship that goes beyond predator-prey. Previous work suggests that archaeological features indicative of social complexity occur suddenly during the latest Epipalaeolithic phase, the Natufian (c. 14,500–11,600 cal BP). These features include sedentism, cemeteries, architecture, food production, including animal domestication, and burials with elaborate mortuary treatments. Our findings from the pre-Natufian (Middle Epipalaeolithic) cemetery of ‘Uyun al-Hammam demonstrate that joint human-animal mortuary practices appear earlier in the Epipalaeolithic. We describe the earliest human-fox burial in the Near East, where the remains of dogs have been found associated with human burials at a number of Natufian sites. This is the first time that a fox has been documented in association with human interments pre-dating the Natufian and with a particular suite of grave goods. Analysis of the human and animal bones and their associated artefacts provides critical data on the nature and timing of these newly-developing relationships between people and animals prior to the appearance of domesticated dogs in the Natufian.

## Introduction

The archaeological record of the Epipalaeolithic period in the southern Levant (ca. 23–11.6 ka cal BP) exhibits considerable variability across time and space. A wealth of archaeological investigations suggest dramatic disparities in material culture between earlier and later phases [1]–[4], explained in terms of pre-adaptive thresholds necessary for the development of subsequent Neolithic farming communities. The Late Epipalaeolithic Natufian culture is well-known for its stone architecture, organized site structures, portable art and decoration, and human burials, some of which display grave goods and personal ornaments. Formalized cemeteries, totalling more than 400 interments, appear for the first time [5]–[9]. These burials document a wide variety of mortuary practices, with treatments of the dead including stone and organic burial containers and installations, worked stone and bone grave goods and, notably, animal inclusions such as early domesticated dog ([10]–[12], although see [13]). Elucidating the social meanings of these variable burial customs is challenging due to limitations of the material culture record and limitations of ethnographic comparisons. However, contextual examination of items accompanying human remains permits preliminary interpretations of this mortuary behaviour. For example, Grossman *et al.* [7] interpret an elderly female Natufian burial containing a unique array of grave goods as the first shaman burial.

Despite early work that seemed to indicate a behavioural break between Early/Middle Epipalaeolithic and later, socially-complex Natufian sites (e.g.,[2]), new excavations suggest an earlier emergence of some of the features characteristic of the Natufian period [14]–[17]. Key features used to differentiate Natufian from preceding Epipalaeolithic groups include the appearance of formalized burial grounds and the origin of mortuary traditions that become characteristic of Neolithic symbolic and ideological life [6]. Two key mortuary practices demonstrate ideological continuity between the Natufian and Neolithic, while highlighting a break between the Natufian and earlier EP groups (e.g., [18]): a) the movement or removal of skulls and, b) special human-animal relationships. For example, the burial of an adult female with a juvenile domestic dog is well-known from ‘Ain Mallaha (‘Eynan) [10], while another human-dog burial comes from Hayonim Terrace [12]. Also, a unique burial from the Late Natufian site of Hilazon Tachtit features an elderly female buried with over 50 tortoise carapaces, several articulated raptor wings, the pelvis of a leopard, and the mandible of a wolf [7]. These unmodified animal parts could be viewed as evidence for changing human-animal interactions or domestication yet-to-come.

‘Uyun al-Hammam is a pre-Natufian burial ground, with elaborate human burials that include evidence for unique human-animal relationships, demonstrating that these features are not unique to the Natufian. The remains of at least eleven individuals, interred in eight graves, represent the earliest known cemetery in the southern Levant and more than double the number of human burials for the entire Early and Middle Epipalaeolithic periods [14]. Recent discoveries at ‘Uyun al-Hammam demonstrate a unique collection (in abundance and diversity) of grave goods and varied patterns of interment associated with the human remains. Two adjacent graves contain the articulated remains of several individuals, and include the following elements: 1) the earliest human-fox burial, 2) the movement of human and animal (fox) body parts between graves and, 3) the presence of red ochre, worked bone implements, chipped and ground stone tools, and the remains of deer, gazelle, aurochs and tortoise – grave goods that together became common among later Natufian and Neolithic burials. The ‘Uyun al-Hammam burials thus demonstrate intriguing human-animal relationships earlier than the first domesticated animal in the region. In light of the significance recently given to the Natufian “shaman” of Hilazon Tachtit [7], our findings provide strong evidence that key aspects of these complex mortuary traditions occurred earlier in the Epipalaeolithic of the Near East than previously thought.

## Analysis

### The Archaeology of ‘Uyun al-Hammam

**Figure 1:**
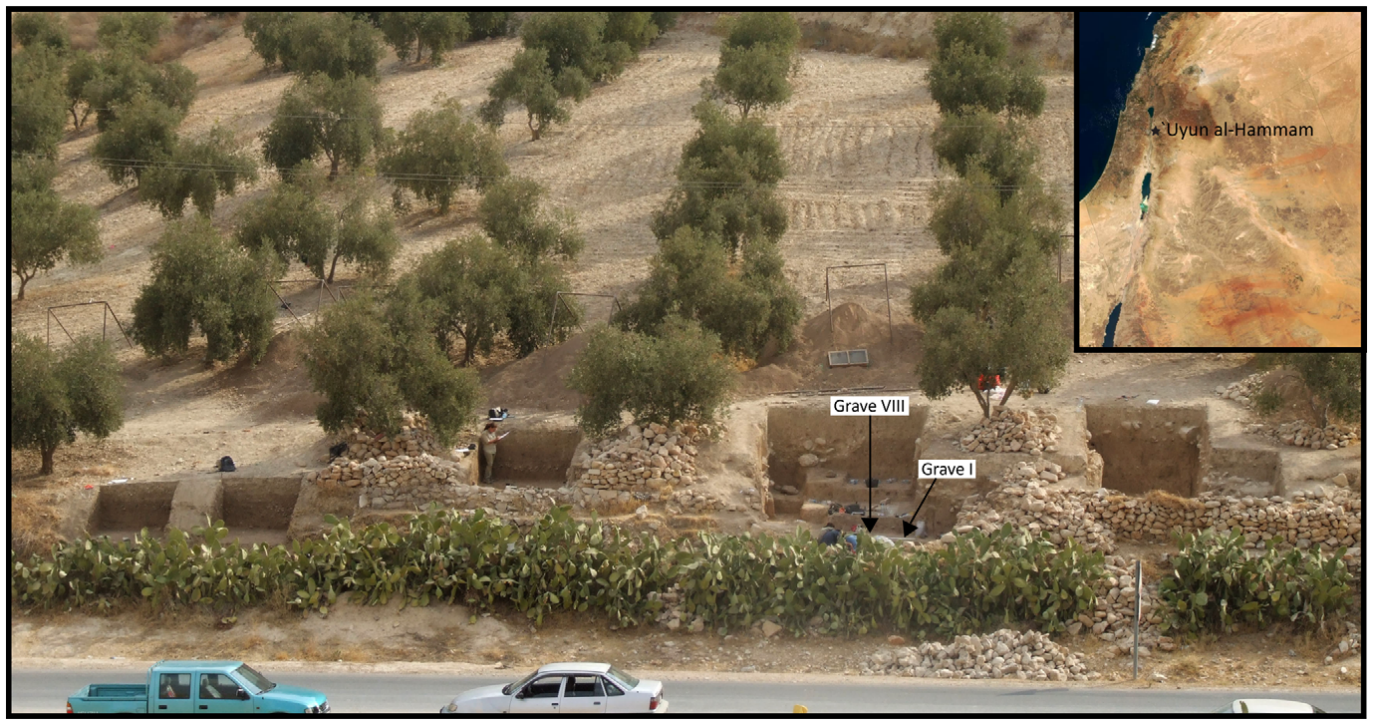
The Middle Epipalaeolithic site of ‘Uyun al-Hammam, with inset showing the location of the site and other Early and Middle Epipalaeolithic sites discussed in the text.

‘Uyun al-Hammam is an open-air site on an ancient river terrace in the small river valley of Wadi Ziqlab, northern Jordan (Figure 1). Situated 200 m a.s.l., the site is between two main geographical features; the Transjordanian Highlands immediately eastwards and the Jordan Valley to the west. Although some portion of the site has been destroyed by recent road-building activity, it extends over an area of approximately 1000–1500 m^2^.

The site's stratigraphy is relatively straightforward and a detailed analysis of the lithic assemblage clearly places the site within the Geometric Kebaran industry of the Middle Epipalaeolithic [19]. The Epipalaeolithic burials all occur within a distinctive Pleistocene palaeosol separated from overlying Holocene colluvium by an erosional unconformity. The uppermost 10–30 cm of the Epipalaeolithic-bearing palaeosol is re-deposited from immediately upslope and represents localised post-occupational erosion that seals all of the burials and occupational horizons. Aside from the burials, occupational features include a potential trampled earth surface, ash dumps, and several discrete refuse middens [19].

**Figure 2:**
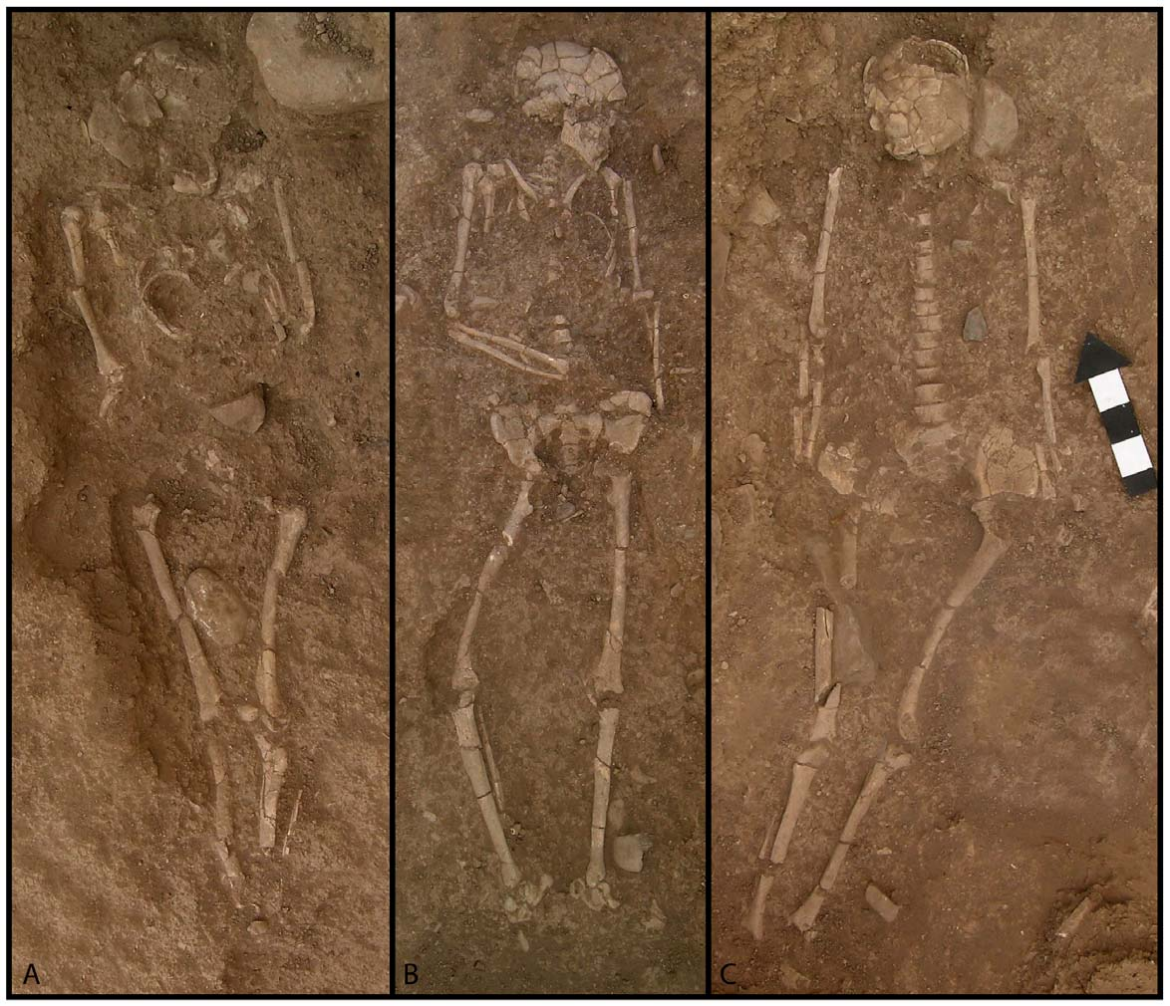
Graves III (A), IV (B), and VII (C) from the Middle Epipalaeolithic cemetery of ‘Uyun al-Hammam.

Graves I to VII were excavated in 2005, and represent the remains of at least nine individuals [14] (Figure 2). In 2008, Grave VI was more fully excavated and revealed the remains of two individuals, and another new grave, Grave VIII, was found. These discoveries more than double the number of known human remains from this time period in the Near East [14]. Some of the burials represent primary interments of single adult individuals (Graves III, IV), while others are secondary burials (Graves II and V) or show reuse of an earlier grave (Graves I, VII and VIII) [14]. None of the individuals show signs of interpersonal violence or obvious pathologies that may indicate cause of death. Although burial pits are not obvious, most of the graves are delimited by body position and the locations of grave goods in contact with the buried individual or large stones around or over the body [14]. Grave goods are present in most of the graves, although the number and type of objects varies. Flint implements, ground stone, red ochre, and partial animal skeletons were found associated with several of the skeletons. Since the burials are dug into pre-existing Epipalaeolithic deposits, the attribution of items as grave goods is conservative—only items in meaningful association or direct contact with the skeletons are included and, therefore, it is possible that we have underestimated the actual number of grave goods in each burial.

Five radiocarbon dates from bone and charcoal within Graves I and VIII as well as an overlying midden deposit (Table 1) bracket use of the site between 17,250–16,350 cal BP and 15,000–14,200 cal BP (95% confidence intervals, using BCal software [20]). This is corroborated by typological analyses of the stone tool assemblage that clearly place all phases within the Middle Epipalaeolithic Geometric Kebaran industry (ca. 17,700–14,750 cal BP)[14].

**Table 1:** Uncalibrated radiocarbon dates from ‘Uyun al-Hammam.

Lab Number	BP	±	Material	Context Notes
TO-11704	12,400	180	Human bone	Burial A, Grave I
OxA-20973	13,650	50	Charcoal	Midden directly overlying Grave VIII
OxA-20974	13,720	55	Charcoal	Abutting fox tail in Grave VIII
OxA-20977	13,785	60	Charcoal	Adjacent to tortoise carapace at same elevation as Grave VIII
OxA-20978	13,685	55	Charcoal	Sediment abutting bone in Grave VIII
Poz-35077	13,700	70	Charcoal	Midden deposit 6 m west of Graves I and VIII

Given the number of individuals interred and complexity of mortuary practices, it is clear that this site represents a pre-Natufian burial ground. The context of the burials and condition of the skeletons suggests that they do not represent one burial episode, but correspond to continued use of the site as a burial ground, either contemporary with or post-dating occupation of the site. A complete analysis of all graves is currently underway and so only the most complicated and spatially related burials, Graves I and VIII, will be discussed in detail here.

### The Human Remains

**Figure 3:**
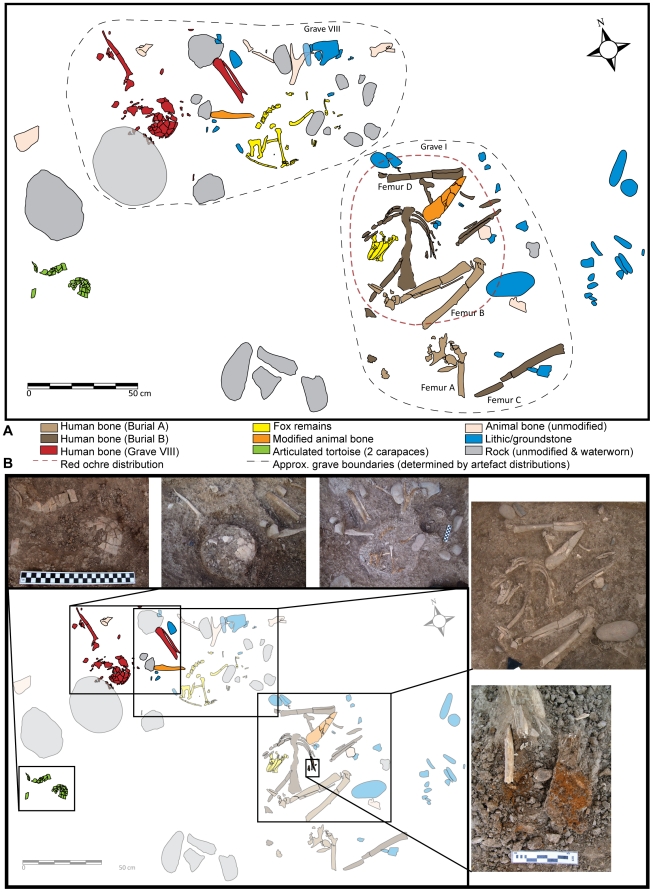
Grave I and VIII showing (A) the distribution of human remains and grave goods in each grave, and (B) blown-up photographs of select grave features.

Grave I included two groupings of bones that we interpret as separate interment episodes identified as Burials A and B (Figure 3). In addition, a variety of miscellaneous bones are consistent with the interpretation of a minimum of two individuals for this grave (Table 2). Burial A includes a fragmentary adult pelvis articulated with lower limb elements, including a left and right femur, a left tibia and articulated bones of a left foot. Burial B includes an articulated but very fragmentary adult thorax, with thoracic vertebrae from T1 to T7, and associated right and left ribs. Among the miscellaneous elements were two adult femora, a left humerus, left scapula, left clavicle, left radius and ulna, which were articulated *in situ*, and all of which are consistent with a single adult individual. Isolated teeth and two mandibular fragments, one relatively robust and one gracile, were discovered in 2000 [21]. The reconstructed size, age and condition of these bones and teeth are also consistent with the other human remains in the grave. We inferred the distinction between Burials A and B on the basis that both appear to be articulated primary interments, with Burial A superimposed upon (and causing the partial disarticulation of) Burial B. The two adult femora that we associate with Burial B are clearly bilaterally paired, and thus belong to a single adult individual.

**Table 2:** Human remains and inferred burial associations from ‘Uyun al-Hammam graves discussed in the text.

Grave I – Burial A	Grave I – Burial B	Grave VIII†
Ossa coxae (Left, Right)^A^ Femur (Right)^A^Femur (Left)^A^Tibia (Left)^A^Calcaneus (Left)^A^Talus (Left)^A^Metatarsal 3 (Left)^A^Metatarsal 2 (Left)^A^ Intermediate Cuneiform (Left)^A^Lateral Cuneiform (Left)^A^Navicular (Left)^A^Mandible*	Thoracic vertebrae^B^Ribs^B^Humerus (Left)*Mandible*Femur (Left)Femur (Right)*Scapula (Left)*Clavicle (Left)*Radius and Ulna (Left)*	Cranium^C^Cervical vertebrae (C1-7)^C^Scapula (Left)^D^Humerus (Left)^D^Radius (Left)^D^Ulna (Left)^D^Tibia (Right)^E^Fibula (Right)^E^

† Grave VIII represents a minimum number of 1 individual, but three clusters of articulated elements may represent up to 3 individuals.

A articulated elements, identified as Grave I, Burial A, a primary interment.

B articulated elements, identified as Grave 1, Burial B, which were directly associated with ochre layer and fox skull and humerus, remains of a primary interment or perimortem movement of torso and skeletal elements.

C articulated elements of cranium and cervical vertebrae, identified as probable male.

D articulated elements of left arm, gracile, identified as probable female.

E articulated elements of right distal limb segment (Fibula and Tibia).

*Possible association of a single individual based upon size/morphology, but no direct articulation, moved after primary interment.

Among the skeletal elements preserved, the pelvis from Burial A provides the only reliable sexually dimorphic traits, and is identified as a probable female on the basis of having broad sciatic notches (category 1, [22]). Discriminant function analysis of osteometric data collected from long-bones, including bone lengths, and diaphyseal and articular dimensions was used to further attempt sex determination (Supporting Information S1). Comparative samples included Levantine Epipalaeolithic skeletons where sex had been determined from pelvic morphology. The discriminant analysis provided ambiguous and male classifications of the two femora of Burial A, while all long bones in Grave I were classified as male. While this is not a conclusive sex determination, given the evidence from the Burial A pelvis, the results suggests that Grave I likely contains interments of one adult female (Burial A) and one adult male (Burial B).

Grave I is taphonomically complex. Subsequent to interment, Burial B appears to have been partially re-opened and another adult individual (Burial A) was interred in the same general area. This burial is represented by a pelvis and articulated lower limbs, which overlie the humerus and vertebral column of Burial B. The thorax, upper limbs and skull of Burial A may have been removed during road construction and subsequent erosion at the terrace edge. Burial A was found with a few small chunks of red ochre (that may have originated from Burial B), and a large limestone pounder lay adjacent to the individual's right femur. Burial B consists of an adult buried together with an articulated fox skull and humerus, a worked bone dagger, chipped and ground stone tools, and unmodified animal bones all lying on a continuous layer of red ochre (see below).

The location of disarticulated remains in Grave I suggests that their movement occurred relatively soon after death or initial interment. During decomposition, joints of the limb generally disarticulate from proximal to distal, while the thorax and spine remain articulated with connective tissue much longer [23]. Excavation of the articulated ribs and vertebrae of Burial B revealed no scapulae underneath. Therefore, if the burial was disturbed after partial decomposition it is plausible that the upper limbs, scapula, clavicle and skull were detached and moved, while the ribs and vertebrae remained intact and ulna and radius articulated. Only upper limb elements from the left side are represented from Burial B, and most extend to the north-east of the thorax. Red ochre underlay the bones in a relatively continuous manner around the rib cage, but occurred more as infrequent chunks around the other isolated bones. Movement of the remains of Burial B after partial decomposition may also explain the missing skull, cervical vertebrae and right arm elements (see below).

Grave VIII is immediately adjacent to Grave I; approximately 10 cm separates objects associated with each grave (excavated in subsequent years, Figure 3). Grave VIII includes both articulated and disarticulated human remains along with a bone spoon or spatula, chipped and ground stone implements, and several unmodified animal bones, including deer antler and a fox skeleton missing only its skull and right humerus (Figure 3).

Human remains included a fragmentary cranium and mandible of an adult with erupted third molars and considerable tooth wear on all molars. The skull featured a prominent glabella and robust left supraorbital margin (categories 5 and 4 respectively, [22]), with prominent superciliary arches. While these suggest the possibility that the skull represents a male, this determination cannot be made with confidence on the current evidence. A large, flat, water-worn stone was brought from the nearby wadi and placed over the skull, while another similar stone was found ∼20 cm to the southwest, adjacent to a wild goat horn core (*Capra sp*.) and several articulated tortoise carapaces (Figure 3). All seven cervical vertebrae were articulated with the base of the skull, but there was 180 degree rotation between atlas and axis, so that the vertebral spines face towards the left, anterior and inferior side of the skull. No other skeletal elements were articulated, suggesting that the skull and cervical vertebrae had been disarticulated from a body and placed here, or moved from another burial (possibly Grave I). While there is no positive evidence that this skull has been moved from Grave I, there is no duplication of cranial or vertebral elements between the graves and many skeletal elements from Burial B (also male) in Grave I are co-mingled, so we cannot rule this possibility out. Grave VIII also included very fragmentary tibia and fibula, in articulation, and a left humerus, scapula, radius and ulna, representing an articulated arm of an adult. Discriminant analysis of humeral metrics suggests that these elements represent an adult female. While the upper limb is situated near the skull, the orientations, different sex determinations, and lack of elements articulating these remains mean that they cannot conclusively be identified as representing the same individual.

### The Earliest Fox-Human Burial

**Figure 4:**
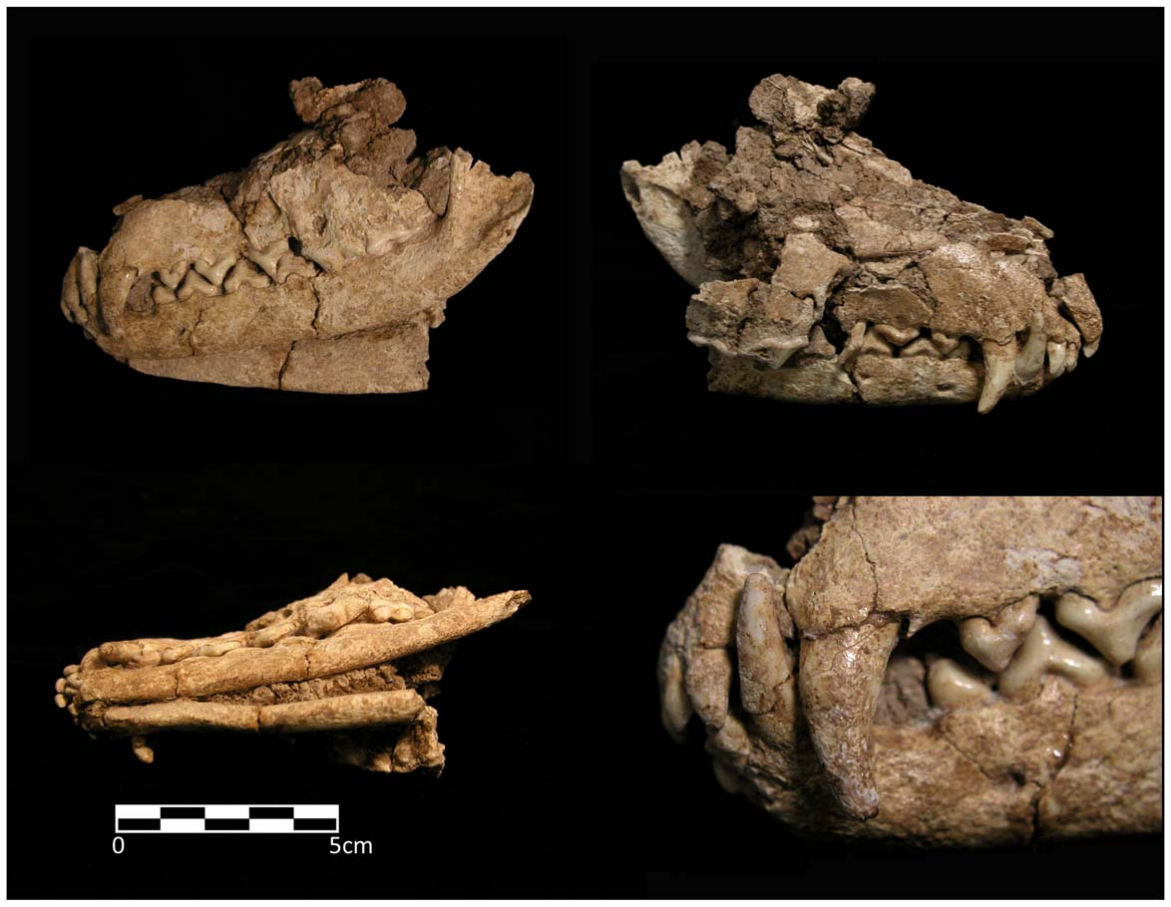
The red fox skull from Grave I after conservation and reconstruction.

Grave I included an articulated fox skull, lying on its right side, underneath the articulated human ribs of Burial B (Figure 3). Although distorted by post-depositional crushing, the skull is remarkably intact (Figure 4). The complete right humerus of a fox was found directly below and adhering to the right side of the mandible. Large pieces of red ochre were found adhering to the underside of the human ribs on both left and right sides, while ochre fragments adhered to the nasal bones, mandible and right side of the fox cranium, as well as in the sediment within the skull cavity. The red ochre here is dense and continuous, suggesting that fox skull and human rib cage were placed on top of a layer of red ochre (Figure 3) as part of the primary interment episode of Burial B.

A morphological comparison was made between the fox skull and 30 skulls representing various canid species, including Pleistocene specimens of Near Eastern canids, archaeological specimens of wolves, foxes and jackals from sites in the Near East, as well as domesticated dogs from Jericho. The closest match based on size and morphology was the Red Fox (*Vulpes vulpes*), a species known to inhabit the area in the late Pleistocene.

A nearly complete fox skeleton, missing its skull and right humerus, was discovered in Grave VIII (Figure 3) adjacent to a bone ‘spoon’, red deer antler, several large flint blades and flakes and several flat unmodified cobbles (see below). The fox skeleton is located 50 cm northwest of the fox skull in Grave I (Figure 3). The size and morphology of the skeleton is consistent with that of the nearby skull. The skeleton has only a left humerus (length = 117.2 mm) while a right humerus (length = 117.0 mm) was found adhering to the skull in Grave I. Considering their proximity, adult status, and the complementary representativeness of elements, we suggest that the skull from Grave I and the post-cranial skeleton from Grave VIII belong to the same animal. The metric similarity of the humeri strongly supports this interpretation.

### Other Grave Inclusions

Although ochre was found in a variety of contexts on-site, the first burial event (Burial B) in Grave I represents the only dense and continuous concentration of red ochre, as both small chunks and as a continuous layer underlying the main portion of the burial (Figure 3). Small chunks of ochre also occurred around the pelvis and femora of Burial A, but in much lower frequencies and only in the areas overlapping with Burial B. Thus, it seems that red ochre is a feature of Burial B, but not necessarily of Burial A. Isolated chunks of red ochre were also found in Grave VIII near the human remains and between the legs of the fox. Ochre has been documented at several other sites in the Levant in association with or staining art objects, human bones, shell, bone and flint [24]–[28].

There are no unmodified stones or cobbles in the Grave I fill. In contrast, Grave VIII contains several water-worn stones. Stone features are known elsewhere on-site [14] and small fire-cracked rocks are found in occupational and midden deposits. Therefore, the absence of stones from Grave I and presence of unusually large and flat cobbles in Grave VIII is likely intentional. In addition, cobbles and large flint flakes were found north and east of the fox skeleton, including five flat stones forming a semi-circular feature (Figure 3).

The inclusion of animal bones in Graves I and VIII is notable. With the exception of an extensive midden, large, complete animal bones are rare from all contexts at ‘Uyun al-Hammam, and horn cores and antler are virtually unknown. Yet Grave I included the fox skull and humerus, the patella from an aurochs, and the remains of gazelle, deer, tortoise, and a notable variety of other species for such a small context (Table 3). Grave VIII contained the fox skeleton, along with red deer antler, a horn core fragment from a wild goat (*Capra sp.*), and other isolated bones. In addition to the disarticulated tortoise carapace fragments recovered from Grave I, the articulated remains of two partial tortoise carapaces were found beside a large cobble near the northwest portion of Grave VIII. Although we cannot clearly associate the tortoise carapaces with Grave VIII (Figure 3), articulated tortoise remains are not found in any other contexts at the site.

**Table 3:** List of faunal remains from Graves I and VIII.

Species Represented	Number of elements (N)
*Cervus cf elaphus*	4
*Dama dama/mesopotamica*	8
*Gazella* sp.	6
*Bos primigenius*	1
*Equus* sp.	1
*Sus scrofa*	3
*Lepus* sp.	1
*Vulpes vulpes* (red fox)	4
Tortoise carapaces	9
*Lagomorpha*, gen. et sp. indet.	1
Land snail	5
Unidentified small mammal	1
Unidentified medium mammal	9
Unidentified large mammal	10
Total	65

List of faunal remains from Graves I and VIII to species, genus or size class (includes faunal remains depicted in Figure 3, except fox skull and skeleton discussed in the text). In Grave I, most of these are isolated faunal remains in the grave fill; whereas Grave VIII did not contain any faunal remains not identified in Figure 3.

**Figure 5:**
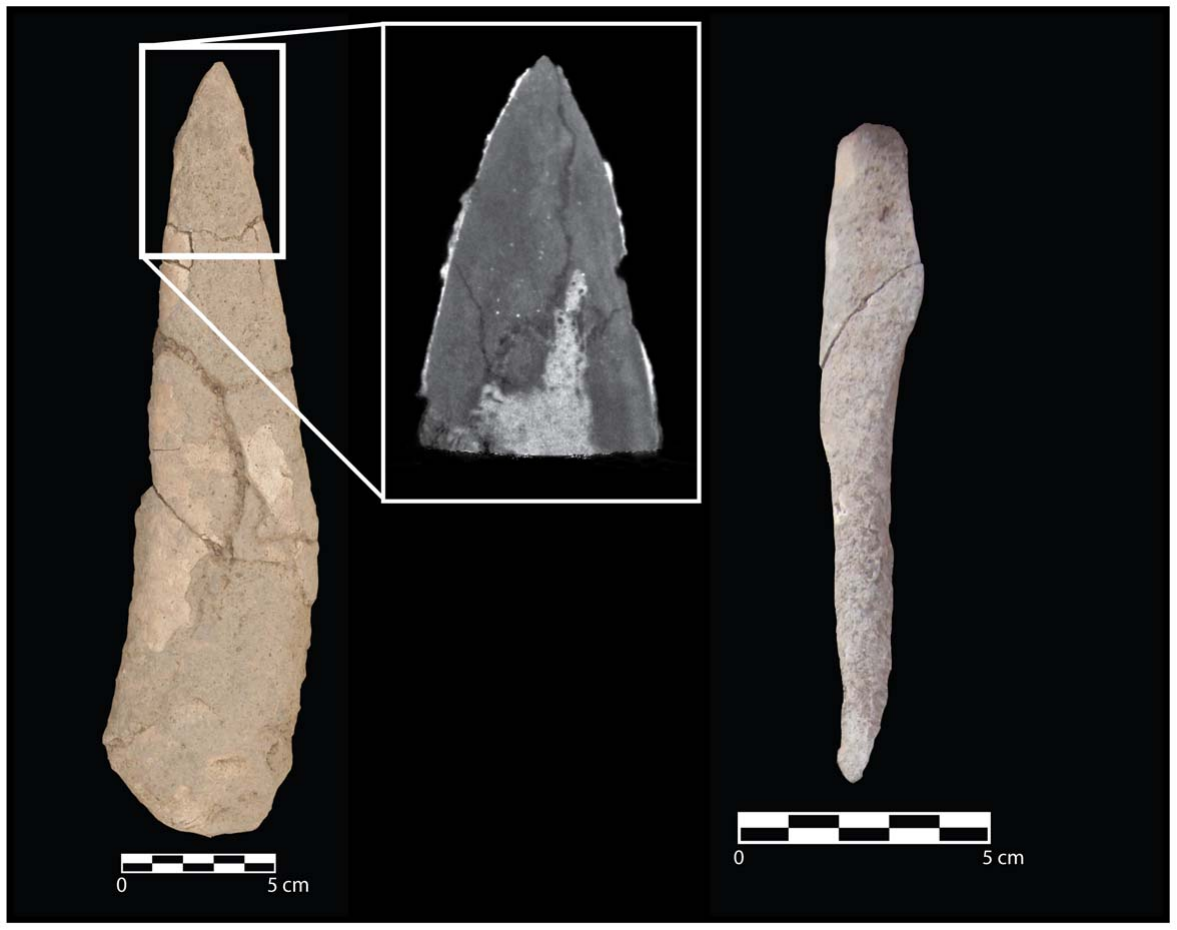
Worked bone objects. (A) Photograph of the bone dagger from Grave I. (B) Micro-CT scan of tip of the bone dagger. (C) Photograph of the bone spoon/spatula from Grave VIII.

Two pieces of worked bone were found in Graves I and VIII: a large bone ‘dagger’ and a flattened spoon/spatula (Figure 5). The dagger (Grave I) has been abraded on one end to form a smooth, rounded termination while the other end was bevelled to a point (Figure 5A). The piece has been heavily altered by human modification and taphonomic processes, including calcium carbonate encrustation, but it is a very close match to a left distal radius from an aurochs (*Bos primigenius*). A microCT scan of the bevelled end reveals differing densities of carbonate and bone that allow a detailed examination of the point's surface topography, including identification of multiple striations running parallel to the long axis of the bone (Figure 5B).

The spoon/spatula from Grave VIII consists of a tibial shaft fragment from a red deer (*Cervus elaphus*) with one end broken at an oblique angle and tapering to a rough point, while the other end has been smoothed to form a shallow depression (Figure 5C). The piece is well-preserved, with minimal taphonomic alterations but was broken *in situ*.

Chipped- and ground-stone implements were found in both graves. Grave I contained a large limestone pounding stone and several trapeze/rectangles associated with Burial A. Several retouched and unretouched flakes, an endscraper, and a small basalt handstone are associated with Burial B. Chipped-stone tools, namely trapeze/rectangle microliths, and cores were also found in the grave fill. Notably, a cache of bladelet cores (n = 3) and endscrapers (n = 7) was found just east of Grave I beside a cylindrical basalt pestle and circular basalt hand stone (Figure 3), but their assignation to this grave cannot be certain. Caches of chipped stone tools are unknown elsewhere at the site and the only complete ground-stone tools come from inside or immediately adjacent to these two graves. In Grave VIII, several large flint blades and endscrapers and a massive flake were found lying directly on a large flat slab immediately north of the fox skeleton (Figure 3).

## Results and Discussion

### The Movement of Human and Non-Human Body Parts

The removal and movement of body parts within and between Graves I and VIII is notable. The missing upper body and skull of Burial A in Grave I is likely the result of erosion. However, the appendicular remains in Burial B were clearly moved post-mortem. The missing skull of Burial B, given the articulated thorax, may have been removed either prior to or during interment of Burial A. The remaining skeletal elements from Burial B are consistent with an adult male individual, as are the cranium and cervical vertebrae from Grave VIII, so it is possible that the skull was removed from Grave I and placed in Grave VIII, or it is simply no longer found in either of these graves.

The human remains of Grave VIII appear to belong to at least two different individuals; removed from two or more corpses relatively early in the decomposition process. The placement of the cranium (covered with a large rock) and a separate, articulated arm beside an articulated fox skeleton and other grave goods is suggestive of intentional interment in a grave rather than a coincidence of grave disturbance.

The removal of skulls is thought to make its first appearance in the Levantine mortuary record in the Natufian (e.g., [5]) but is documented from many Neolithic graves as a symbolic act [8], [18], [29], [30]. We have one other instance at ‘Uyun al-Hammam of a secondary grave consisting only of a human skull and some mammalian long bones [14], so skull removal and reburial at ‘Uyun al-Hammam demonstrates that this burial practice originated prior to the Natufian.

### Fox Remains in Burial Contexts

The movement of the fox skull and humerus from Grave VIII to Grave I is clearly significant. We suggest that, rather than the fox being treated as a ‘grave good’ (e.g., personal adornment) it had a special relationship (i.e., companion) to the humans in these graves. Just as the skull of Burial B was removed during a later disturbance of this grave, and a skull placed into Grave VIII, the fox skull was removed from Grave VIII and re-buried with an individual in Grave I. It is possible that the link between fox and human was such that when the human died the fox was killed and buried alongside. Later, when the graves were re-opened, these links were remembered and bones moved so that the dead person would continue to have the fox with him or her in the afterlife.

Foxes appear in faunal assemblages from the Palaeolithic and increase in frequency to become especially abundant in the Late Natufian [31]. However, most sites exhibit disarticulated and fragmented fox bones, sometimes burnt or with butchery marks, and treated in a manner similar to other prey animals [31]. Fox remains occur in other contexts at ‘Uyun al-Hammam, including middens, and their consumption is quite likely. However, the only articulated animal remains and the only relatively complete animal skeleton from the site is the fox from Graves I and VIII. In this context it is clear that these fox remains are not related to consumption or exploitation of some secondary product, such as a pelt.

Natufian burials at ‘Ain Mallaha [32] and Hayonim Cave [33], [25] contain isolated fox mandibles or, more commonly, perforated teeth. In the Neolithic, foxes are common, particularly at the ritual burial site of Kfar HaHoresh where fox mandibles are found associated with human skulls and partially articulated fox remains are known from two child burials [8], [47], [34]. Here they are interpreted as symbolic within a Neolithic mortuary tradition that has its roots in the Natufian [30]. The symbolic significance of foxes is also attested by their frequent occurrence as motifs on the T-shaped pillars and elsewhere at Göbleki Tepe [35]. At ‘Uyun al-Hammam it seems the fox was viewed and treated as ideologically different from other animals. Like the humans, the fox was buried complete, associated with red ochre, and had its head removed and moved elsewhere (with another burial). The fox was never domesticated, but its increased prominence in mortuary, as well as domestic, contexts with the Neolithic makes its meaning even more elusive.

Other complete and articulated animal skeletons within burial contexts are extremely rare from Epipalaeolithic and Neolithic sites. Some of the earliest examples are domestic dogs from Israel [10]–[12]. In the Early Natufian levels at ‘Ain Mallaha a woman was buried with her hand on a puppy placed above her head [10]. From the Late Natufian layers at Hayonim Terrace, three humans (H7, H8, and H10) were buried with two dogs in a pit also containing tortoise shells and large cobbles placed over some of the bones [12]. Although dog domestication appears to have begun outside the Near East ([36]–[38], although see [39]), recent work suggests that small dogs originated from gray wolves here [40]. Considering that the earliest domestic dogs in the Near East are small [10]–[11], it is not much of a stretch to think that similarly-sized foxes could have been considered as potential domesticates to prehistoric people. Despite evidence for dog domestication in the Natufian, the presence of this fox in two adjacent burial contexts suggests a complex relationship between humans and foxes earlier in the Epipalaeolithic. Although canid remains, including those of wolf, have been found in other contexts at the site [41], none are complete, well-preserved, articulated, or come from other discrete burial contexts. The dog domestication process likely occurred symbiotically, initially through wolves and humans living in close proximity ([42]–[43], [44] and references therein, [37], [45] and references therein) and may have been primarily related to hunting [46]. Studies have shown that foxes are easy to tame [47], and share many sensory and other features with wolves, which might make them amenable to domestication. They are smaller and easier to control – although more skittish and timid – than the wolf. It seems likely that foxes could have shared a similar type of relationship with humans as wolves did, even if they were never truly domesticated. Although we may never be able to reconstruct the nature of the relationships between humans and early domesticated dogs (were they pets or work animals, or both?), their inclusion in burials reveals ties of emotional consequence. It is possible that the burial of a fox with a human might have had the same social, ideological or symbolic significance as that of a human with a dog (i.e., [48]).

Animals are clearly a significant feature of Natufian and later Neolithic burial practices [8], [18], [30]. Furthermore, the animals represented in these graves are from a small, specific range of species, including aurochs, fox, tortoise and gazelle and, thus likely had a special relationship to the individuals interred or doing the interring. It seems these animals were more than just economic resources from which food and other items could be taken and modified into tools, clothing, or decorations (see below).

### Summary

Several features of the ‘Uyun al-Hammam burials link them to later burial practices include the disturbance and co-mingling of humans and animals, the presence of several interments and re-use of graves, secondary burial, the removal of human skulls, the use of red ochre; grave goods, and the burial of a fox with a human (e.g., [6]). The Middle Epipalaeolithic burials discussed here clearly belong to a formalized burial ground and the graves exhibit complex and elaborate treatments of the dead. Prior to the discoveries at ‘Uyun al-Hammam, few burials dating to this period have been found in the southern Levant [16] (Table 4). Thus, the importance of the burials at ‘Uyun al-Hammam goes beyond simply their rarity and the richness of represented mortuary treatments. The burials, and similarities in mortuary practices demonstrated between ‘Uyun al-Hammam and the succeeding Natufian and Neolithic has central bearing on broad cultural developments in the region. Specifically, these burials suggest cultural continuity in the region that stretches from the Last Glacial Maximum (ca. 18,000 cal BP) into the Neolithic some 10,000 years later. This continuity is even more striking as it extends over a period of massive social, technological, economic and ideological change. Before this discovery, it was possible to argue a cultural break between the mobile hunter-gatherer traditions of the Early/Middle Epipalaeolithic and the sedentary ‘socially-complex’ predecessors of Neolithic farmers. Now, the cultural linkage in mortuary practices between Early/Middle and Late Epipalaeolithic groups requires that we look to the full range of factors that drove the development of social change in the southern Levant, rather than attributing these developments to some kind of cultural or ideological break. Not surprisingly, non-economic connections between people and animals existed throughout the Epipalaeolithic, and likely earlier. They pre-date animal domestication and are reflected in human burials.

**Table 4:** Summary of Early and Middle Epipalaeolithic burials from the southern Levant [16], [49]–[54].

Site	Age	Burial Description
Ein Gev I (Israel)	19,000 cal BP	A single, primary interment of an adult female below a living floor with three associated gazelle horn cores
Ohalo II (Israel)	22,500 cal BP	Single primary interment of an adult male with three stones and an incised bone behind his head and a hammerstone between his legs
Kharaneh IV (Jordan)	∼19,000 cal BP	Single, primary interment of an adult male with two gazelle horn cores above his skull and a large cobble over his pelvis; second partial adult male burial
Ayn Qasiyyah (Jordan)	19,800–20,400 cal BP	Single, primary interment of an adult male, tightly bound and placed in a sitting position
Neve David (Jordan)	15,000–16,000 cal BP	Single primary interment of an adult male buried with a three ground stone tools; grave marked by large stones; second partial burial
Qadish Valley (Lebanon)	17,500–14,500 cal BP	Single, primary interment of an adult male in a well-defined pit with two polished stone pebbles
Wadi Mataha (Jordan)	17,000 cal BP	Single primary interment of an adult male buried bound and face down with a breached ground stone bowl and flint blade

## Supporting Information

Supporting Information S1 **Discriminant analysis to determine sex.**
(DOC)
