# Supplementary material for: A Unique Human-Fox Burial from a Pre-Natufian Cemetery in the Levant (Jordan)
Source: PLoS One. 2011 Jan 26;6(1):e15815. doi: 10.1371/journal.pone.0015815 (PMC3027631; doi:10.1371/journal.pone.0015815)
Supplement: Supporting Information S1 — Discriminant analysis to determine sex. (DOC) [file pone.0015815.s001.doc]

**Supplementary Information Tables S1**

**Discriminant analysis to determine sex.** Well preserved postcranial skeletal elements in each burial feature were used, in part, to identify which remains we consider to be representing interment events. Better preserved elements were used to estimate the sex of individuals represented within the graves, using discriminant function analysis. Within Grave I, Burials A and B, morphometric characteristics of long-bones were compared to metric variation among Late Epipalaeolithic (Natufian) remains from the sites of Hayonim, Nahal Oren, Ayn Mallaha, Rakefet, as well as Early and Middle Epipalaeolithic remains from Ohalo II, and Wadi Mataha, respectively. The human remains included from these sites represent complete skeletons where sex was identifiable on the basis of sexually dimorphic characteristics of the ossa coxae or the skull. Further discriminant analysis was conducted on the morphology of the left humerus from Grave VIII. The utility of these discriminant analyses is somewhat limited by small sample sizes and the small number of variables available for comparison, however they provide the only means of estimating the sex of the isolated bones within the graves. As such, the analyses should be viewed as tentative sex determinations.

The classification results and discriminant statistics are presented below. While long bone metrics of Grave I Burial A suggest more male morphology, the broad sciatic notch of the articulated pelvis suggests that this is probably a female. The disrcriminant analysis was conducted largely on biomechanically plastic features of the diaphyses. This would be inflated by high levels of mobility, and may explain the ambiguous discriminant results. It is most appropriate to consider this burial as probable female with a relatively low level of confidence on account of the fact that the pelvis preserved the sciatic notch, a less reliable indicator, as the only observable sexually dimorphic trait. All measurable long-bone elements which we have grouped into Grave I 'Burial B' are classified as Male. This provides support for the interpretation that these remains belong to one individual, an adult male, although the bones have been disarticulated. The humerus interred with the articulated arm in Grave VIII is very gracile, and classifies as morphologically similar to Natufian females. The cranium in this Grave has 'male' characteristics of prominent glabella and supercilliary arches, and a thick superorbital margin. As a result, the remains in Grave VIII may represent either a single individual of ambiguous sex determination, or multiple individuals including an adult male and adult female.

Table S1A. Summary of discriminant sex classifications for long-bones.

|  | **Grave I -** *Burial A* | **Grave I -** *Burial B* | **Grave VIII** | **Statistics** |
| --- | --- | --- | --- | --- |
| Clavicle | NA | Predicted Group = Male, P=.936 (Strong) | NA | Table S2 |
| Humerus | NA | Predicted Group = Male, P=.663 (Weak) | Predicted Group = Female, P=1.000 (Strong) | Table S3 |
| Radius | NA | Predicted Group = Male, P=.986 (Strong) | NA | Table S4 |
| Ulna | NA | Predicted Group = Male, P=.948 (Strong) | NA | Table S5 |
| Femur | Right Predicted Group=Male  P=.997 (Strong)  Left Predicted Group=Male  P=.506 (Ambiguous) | Right Predicted Group=Male  P=.984 (Strong)  Left Predicted Group=Male  P=.995 (Strong) | NA | Table S6 |
| Estimated Sex | Probable Female / ambiguous (see notes in text) | Probable Male | Cranium = Probable Male  Humerus = Probable Female |  |

###### Table S1B. Discriminant Statistics Structure Matrix, Clavicle

|  | **Function 1** |
| --- | --- |
| Maximum length | 0.966 |
| Maximum diameter 50% | 0.494 |
| Minimum diameter 50% | 0.196 |
| **Significance** | **---** |
| Eigenvalue | 1.394  100.0% |
| Canonical Correlation | 0.763 |
| Wilk’s Λ (before function) | 0.418 |
| Chi-square (of Wilk’s Λ) | 8.728 df=6, p=0.189 |

###### Table S1C. Discriminant Statistics Structure Matrix, Humerus

|  | **Function 1** |
| --- | --- |
| Maximum diameter 35% | 0.821 |
| Minimum diameter 35% | 0.713 |
| Maximum diameter 50% | 0.699 |
| Minimum diameter 50% | 0.681 |
| Maximum length | 0.572 |
| Supra-olecranon A-P diameter | 0.433 |
| **Significance** | **---** |
| Eigenvalue | 1.394  100.0% |
| Canonical Correlation | 0.763 |
| Wilk’s Λ (before function) | 0.418 |
| Chi-square (of Wilk’s Λ) | 8.728 df=6, p=0.189 |

###### Table S1D. Discriminant Statistics Structure Matrix, Radius

|  | **Function 1** |
| --- | --- |
| Minimum diameter 50% | 0.941 |
| Maximum length | 0.752 |
| Maximum diameter 50% | 0.484 |
| **Significance** | **---** |
| Eigenvalue | 0.923  100.0% |
| Canonical Correlation | 0.693 |
| Wilk’s Λ (before function) | 0.520 |
| Chi-square (of Wilk’s Λ) | 6.212 df=3, p=0.102 |

###### Table S1E. Discriminant Statistics Structure Matrix, Ulna

|  | **Function 1** |
| --- | --- |
| Maximum length | 0.844 |
| Maximum diameter 50% | 0.742 |
| Minimum diameter 50% | 0.354 |
| **Significance** | **---** |
| Eigenvalue | 1.406  100.0% |
| Canonical Correlation | 0.764 |
| Wilk’s Λ (before function) | 0.416 |
| Chi-square (of Wilk’s Λ) | 9.218 df=3, p=0.027 |

###### Table S1F. Discriminant Statistics Structure Matrix, Femur

|  | **Function 1** |
| --- | --- |
| Subtrochanteric Max Diameter | 0.771 |
| Maximum Length | 0.481 |
| 50% medio-lateral diameter | 0.455 |
| 50% minimum diameter | 0.436 |
| 50% maximum diameter | 0.412 |
| 50% antero-posterior diameter | 0.389 |
| Subtrochanteric Min Diameter | 0.236 |
| **Significance** | **---** |
| Eigenvalue | 3.704  100.0% |
| Canonical Correlation | 0.887 |
| Wilk’s Λ (before function) | 0.213 |
| Chi-square (of Wilk’s Λ) | 16.259 df=7, p=0.023 |
